# Functional Feed Assessment on *Litopenaeus vannamei* Using 100% Fish Meal Replacement by Soybean Meal, High Levels of Complex Carbohydrates and *Bacillus* Probiotic Strains

**DOI:** 10.3390/md9061119

**Published:** 2011-06-17

**Authors:** Jorge Olmos, Leonel Ochoa, Jesus Paniagua-Michel, Rosalia Contreras

**Affiliations:** Molecular Microbiology Laboratory, Department of Marine Biotechnology, Centro de Investigación Científica y de Educación Superior de Ensenada (CICESE), Ensenada, B.C., Mexico; E-Mails: leonel.ochoa@gmail.com (L.O.); jpaniagu@cicese.mx (J.P.-M.); rcontre@cicese.mx (R.C.)

**Keywords:** *Bacillus*, probiotics, shrimp, functional feeds, soybean meal

## Abstract

Functional feed supplemented with alternative-economic nutrient sources (protein, carbohydrates, lipids) and probiotics are being considered in shrimp/fish aquaculture production systems as an option to increase yield and profits and to reduce water pollution. In this study the probiotic potential to formulate functional feeds have been evaluated using four dietary treatments: Treatment 1 (B + Bs); *Bacillus subtilis* potential probiotic strain was supplemented to a soybeanmeal (SBM)—carbohydrates (CHO) basal feed. Treatment 2 (B + Bm); *Bacillus megaterium* potential probiotic strain was supplemented to the same SBM-CHO basal feed. In Treatment 3 (B); SBM-CHO basal feed was not supplemented with probiotic strains. Treatment 4 (C); fishmeal commercial feed (FM) was utilized as positive control. Feeding trials evaluated the survival, growth, and food conversion ratio and stress tolerance of juvenile *Litopenaeus vannamei* (Boone) Pacific white shrimp. Best overall shrimp performance was observed for animals fed with Treatment 1 (B+Bs); additionally, stress tolerance and hemolymph metabolites also showed the best performance in this treatment. SBM-CHO basal feed not supplemented with probiotic strains (B) presented smaller growth and lower feed conversion ratio (FCR). Shrimps fed with the fishmeal commercial feed (C) presented the lowest stress tolerance to high ammonia and low oxygen levels. Specifically selected *B. subtilis* strains are recommended to formulate functional and economical feeds containing high levels of vegetable; protein and carbohydrates as main dietary sources in *L. vannamei* cultures.

## 1. Introduction

Global population demand for aquatic food products is growing in importance; however fisheries capture production has leveled off and most of the main fishing areas have reached their maximum potential [1]. Due to this, fishmeal prices have increased considerably in recent years. In addition, concern has also arisen about the negative impact of fishmeal production on global fisheries ecology and on the environment [2,3]. Aquaculture, probably the fastest growing food-producing sector, presents the greatest potential to meet demands for aquatic food supply. However, in order to accomplish these goals, the sector will face significant challenges to increase aquaculture profitability.

Cultured species depend on our knowledge of nutrition, biochemistry, physiology and genetics, among others. Feeding represents 40 to 60% of total production cost in shrimp farms; therefore, new varieties of feed formulations must be directed to be well-balanced and inexpensive diets [4]. Use of animal protein sources, such as fishmeal in shrimp feeds, is expected to be considerably reduced as a consequence of increasing economical, environmental and safety issues [5–8]. Partial or complete fishmeal substitution of by-product meals by vegetable protein and carbohydrate sources is a major concern to the field [9,10].

Soybean meal (SBM) has been one of the most studied ingredients to substitute fishmeal (FM) in aquatic animal feeds; however unsatisfactory results have been obtained due to SBM containing toxic-antinutritional ingredients to monogastric animals [11,12]. In addition, soy protein concentrate (SPC) is difficult to produce and expensive for shrimp feed formulation. High levels of complex carbohydrates also represent an important obstacle to shrimp aquaculture, due to shrimp’s limited carbohydrates digestion capabilities [13–15].

Functional foods are defined as “foods with dietary ingredients that provide healthy and economical benefits beyond basic nutrition”. Probiotic bacteria, supplemented in functional feeds, could transform aquaculture in a sustainable, competitive and profitable industry [10,14]. A probiotic is defined as a living microbial supplement that: (a) positively affects hosts by modifying the host-associated microbial community and immune system; (b) secrete a variety of enzymes to improve feed degradation enhancing its nutritional values; and (c) improves quality of environmental parameters [10,16–18].

*Bacillus* probiotic strains have been used to improve *L. vannamei* growth performance, digestive enzyme activity and the immune response, getting good results in all the parameters measured. However, the 100% replacement of FM by SBM and high levels of complex carbohydrates (CHO) has never been done successfully, with and without *Bacillus* strains. The maximum amount of SBM included in *L. vannamei* experimental diets without health problems reach 20%, but always in combination with 20% or more of FM inclusions [19–21].

The present study evaluated a functional feed without FM, but supplemented with high levels of SBM, high levels of complex CHOs and a *Bacillus subtilis* probiotic strains. Survival impacts, growth and performance and immune parameters of *Litopenaeus vannamei* were evaluated and excellent results were obtained, opening great opportunities to new shrimp and fish feed formulations.

## 2. Experimental Section

### 2.1. Feeds Preparation and Analysis

Formulations were developed with NUTRION Software containing 28% crude protein and 7% lipids. Macro-ingredients (48% soybean meal, 19% corn flour, 16% wheat flour, 2% algae meal, and 2% corn gluten and 2% wheat gluten) were thoroughly mixed, vitamin and minerals mixture was then incorporated gradually. Oil-based ingredients (fish oil 3%, soybean oil 3% and lecithin 2%) and 30% water was then added to obtain the final consistency. Feeds were supplemented with *B. subtilis* and *B. megaterium* containing both formulations approximately 1.2 × 10^4^ CFU/g (1g/Kg) inclusion. A meat grinder of 1.6 mm diameter holes was used to pellet production at temperatures between 70 and 75 °C. Spaghetti-like strings produced were air dried in a vent hood at room temperature overnight. Five minutes treatment at 95 °C was applied to the pellets to test the probiotic temperature resistance. Feeds were broken up, sieved to a convenient pellet size and used to evaluate shrimp behavior. Functional feed proximal analyses are shown in Table 1. Treatment 1 (B + Bs); *Bacillus subtilis* potential probiotic strain was supplemented to soybean-meal (SBM)-carbohydrates (CHO) basal feed. Treatment 2 (B + Bm); *Bacillus megaterium* potential probiotic strain was supplemented to the SBM-CHO basal feed. In Treatment 3 (B); SBM-CHO basal feed was not supplemented with any probiotic strain. Treatment 4 (C); fishmeal commercial feed (FM) was utilized as positive control. *Bacillus* potential probiotic strains viability was established through microbiological and molecular methods [10,22,23].

### 2.2. Shrimp Growth Conditions

Shrimp were purchased from a local hatchery dealer; acclimatized in two fiberglass tanks of 2.5 m^3^ each, for a period of 20 days prior to the experiment. Three tanks of 120 L were used for each treatment, containing 30 juvenile shrimps with an average initial weight of 5.83 g ± 0.2 g randomly stocked in each tank. Shrimps were fed to satiation three times per day at 08:00, 14:00 and 22:00 hours, throughout a four weeks period. Feeding was adjusted daily according to ingested rate to be sure feed was totally consumed. Before feeding, molts, feces and dead shrimps were removed and 10% of water in each tank was exchanged daily by new sea water. Feed intake, mortality and water quality parameters were recorded daily. At the end of the trial survival rate was evaluated. Shrimp’s weights were determined at the beginning (Initial Weight) and at the end (Final Weight) of the experiment. Weight gain and feed consumption were used to calculate several parameters; Feed conversion ratio (FCR) = feed consumed (dry weight)/live weight gain (wet weight). Daily Weight Gain (DWG; g/day) was calculated as: (Final weight (g) − Initial weight (g))/days.

### 2.3. Hemolymph Parameters Measurement

At the end of the trial, twelve shrimps from each treatment were selected and hemolymph parameters were evaluated. Shrimps were bled and 0.5 mL individual samples were obtained from the pericardial cavity using a 1.3 mL syringe containing 1.0 mL of SIC-EDTA anticoagulant (NaCl 450 mM, KCl 10 mM, HEPES 10 mM, EDTA 10 mM; pH 7.3). Samples were place in 5 mL crystal tubes 1.5 mL Eppendorf tubes with anticoagulant and centrifuged at 800 × g for 10 min. Serum Plasma was collected and held at 4 °C; glucose, lactate, and total cholesterol, were measured using ATAC 8000 Equipment Inc. In addition, a 50 μL sub-sample was separated from the beginning to circulating hemocyte count (THC) determination.

### 2.4. Stress Parameters Evaluation

To evaluate shrimp stress response capabilities acquired throughout feeding, 60 mg/L of ammonia was added to each of the 120 L tanks at the end of the trial. Seawater was maintained at 35 ppt (parts per thousand) and 28 °C and survival was evaluated during 24 h time tests. Oxygen test was run by the static method using seawater at 35 ppt and 28 °C, stabilized at 6.70 mg/L O_2_. Twelve shrimps (12 ± 0.16 g) from each treatment were placed in 38 L aquaria by triplicate and oxygen was not supplemented during 24 h time test. Oxygen LD_50_ Percentage survival rate was evaluated and the final survival was reported for each treatment.

### 2.5. Statistical Analyses

Experimental units (tanks and aquariums) were distributed on a completely randomized way. Quantitative data were checked for normality and homoscedasticity. Data were analyzed by using one way analysis of variance (ANOVA), to search for significant (*p* < 0.05) differences among treatment means [24]. Duncan’s and Tukey’s multiple comparisons of means were carried out with the Statistica V6 program.

## 3. Results

### 3.1. Functional Feed Analysis

The proximal composition of SBM-CHOs basal feed (B) and FM commercial feed (C) utilized in this work are shown in Table 1. Protein percentage almost reached a 10% difference between both feeds, containing higher levels the fishmeal diet (36.39%) than the basal feed (27.41%). In addition, it is important to point out that the basal feed protein source was SBM.

However, carbohydrates concentration was higher in basal feed (49.50%) than in fishmeal formulation (38.40%), exceeding recommended levels to shrimp feeds [25].

**Figure 1:**
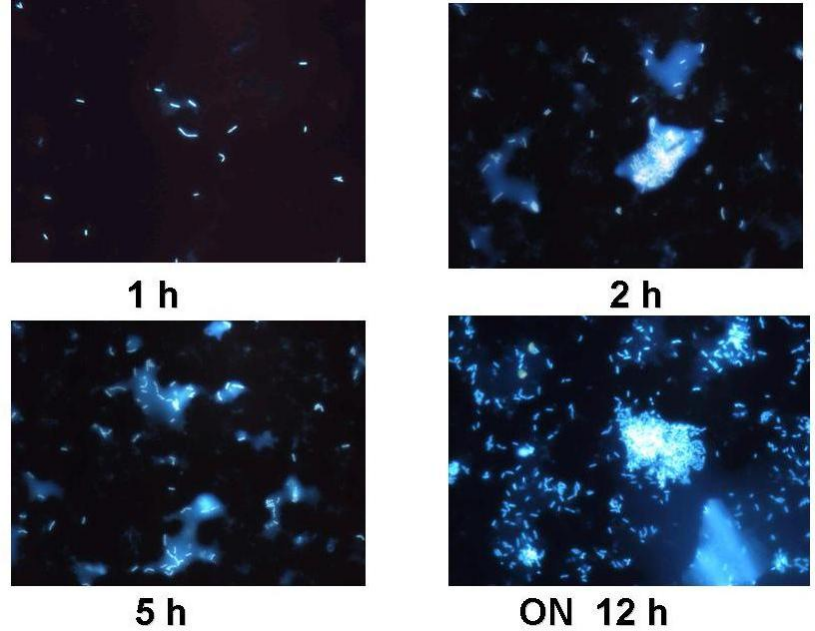
Kinetic growth and DAPI stained cells of *Bacillus subtilis* recovered from B + Bs treatment.

**Figure 2:**
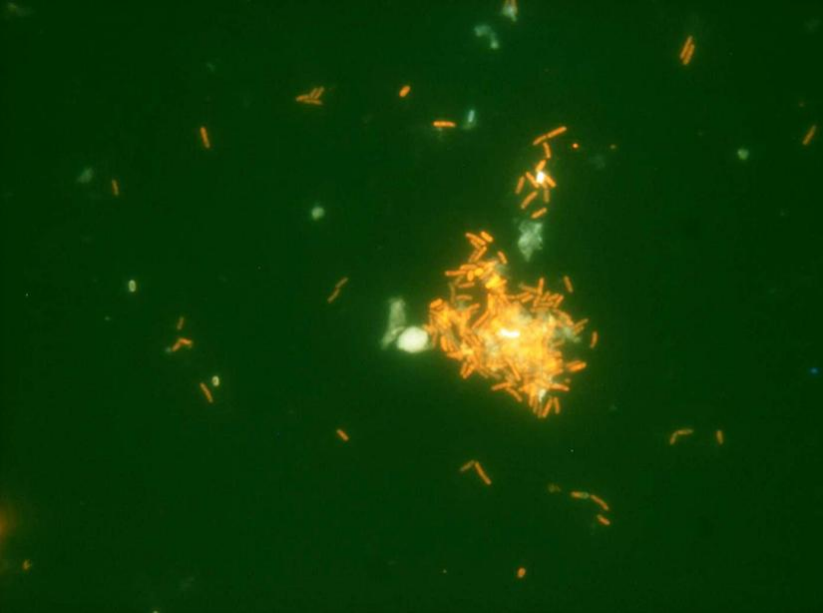
Fluorescent *in situ* Hybridization (FISH) of feed particles of B + Bs treatment.

Bacteria were supplemented at the same concentration for Treatment 1 (*B. subtilis*) and Treatment 2 (*B. megaterium*)(*P* > 0.05), containing both formulations approximately 1.2 × 10^4^ CFU/g. After a year, feeds did not show any considerable variation in bacterial concentration and viability. *B. subtilis* strain was recovered from B + Bs feed and DAPI stain cells methodology was accomplished for probiotic detection and quantification (Figure 1) [22,23]. Furthermore, B + Bs isolated *B. subtilis* strain was grown until middle exponential phase in Shaeffer’s sporulation medium to perform Fluorescent *in situ* Hybridization (FISH) (Figure 2), using low G + C specific probe for cell viability quantification [23,26]. In addition, bacterial identification by PCR using *Bacillus* specific oligonucleotides was carried out [14]. DNA samples from B + Bs feed isolated bacteria amplified a 640 bp expected fragment size, from the *16S rDNA* gene of *B. subtilis* (data not shown).

### 3.2. Shrimp Growth

Throughout the feeding trials water temperature was maintained at 28.5 ± 2.22 °C, 35.2 ± 1.19 ppt salinity and 6.7 ± 0.20 mg/L of oxygen concentration. Total ammonium 0.1 ± 0.07 mg/L; nitrite 0.03 ± 0.01 mg/L and pH 7.6 ± 0.46; were set within acceptable ranges [27]. Statistically significant differences within parameters were not observed. Shrimps initial weight was not different among the treatments. However, at the end of the trial organisms fed with Treatment 1 (B + Bs) reached the greatest growth performance. Additionally, they did exhibited significant differences with respect to the other treatments (Table 2). It is important to remember that Treatments 1, 2 and 3 were formulated with vegetable protein sources (Experimental Section). In this sense, *B. subtilis* strain utilized produce enzymes to hydrolyze feed ingredients from vegetable origin [10].

Food Conversion Ratio (FCR) was statistically different between treatments, showing the best results in shrimps fed with Treatment 1, where *B. subtilis* strain had been included (Table 2). SBM-CHOs basal feed (B) exhibited the FCR higher values (2.49 ± 0.15) as we expected, due to high levels of vegetable ingredients and non probiotic bacteria inclusion (Table 2). SBM-CHOs basal feed + *Bacillus megaterium* (B + Bm) showed less capabilities to digest vegetable ingredients than *B. subtilis* did. However, Treatment 2 shrimps still obtained better FCR than basal feed treatment (B) and no FCR differences were shown with respect to FM commercial feed treatment (C) (Table 2). Shrimp survival rate ranged from 96 to 100%, showing no significant differences among treatments (*p* > 0.05). However, shrimps fed with Treatment 1 exhibited a higher survival rate (Table 2).

### 3.3. Hemolymph Metabolites Quantification

Serum Plasma glucose levels, lactate and total cholesterol were positively affected, principally those presented by shrimps from Treatment 1 (Table 3). Feeds formulation and growth environmental conditions are dominant factors affecting hemolymph metabolites concentration and shrimp performance [28]. In this research, hemolymph metabolites were up-regulated by supplementing a vegetable feed formulation with a *B. subtilis* probiotic strain (Table 3). High glucose, lactate and cholesterol levels obtained with *B. subtilis* diet (B + Bs), were significantly different with respect to the other treatments (*p* < 0.05). Furthermore, results obtained with Treatments 2, 3 and 4, did not exhibit significant differences between them (*p* > 0.05). Total hemocyte counts (THC) were also positively affected in Treatment 1 and were significantly different (*p* < 0.05) from the other treatments (Table 3). Recent studies provide evidence that greater amounts of dietary protein levels improve immune system functionality, increasing shrimp’s hemocytes concentration [15]. However, in this study Treatment 1 shrimps with 27.41% vegetable protein level have reached greater hemocytes counts than Treatment 4 that contains 36.39% animal protein. SBM protein could be an obstacle to protein digestion and assimilation, due to its anti-nutritional compounds [9,29].

Pascual and coworkers also demonstrated the CHOs digestion deficiency from cultivated shrimps. In this sense, low levels of shrimps carbohydrolases is a conditional deficiency from using high amounts of complex carbohydrates, like starch [13,14,25]. However, in this work we demonstrated that using the appropriate probiotic bacteria; protein and carbohydrates origin and levels seems to be not so important.

### 3.4. Stress Condition Evaluation

**Figure 3:**
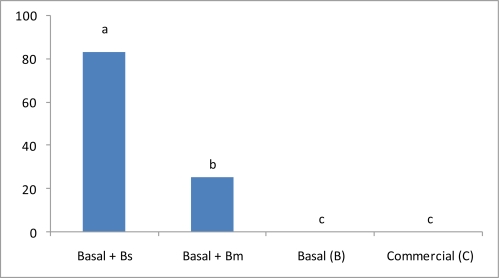
Y-axis represents percentage survival rates of *L. vannamei* during and after ammonia. Semi-static method was tested on the different treatments. Means in the same row with different superscripts are significantly different (*P* < 0.05).

Surprising results were obtained with the ammonia test evaluation. Survival rate was significantly affected and only Treatment 1 shrimps presented good survival results (Figure 3). In this respect, it is conclusive that functional feeds promote response capabilities improving shrimps health status and performance. Treatments without probiotic strains had 100% mortalities in 24 h in the ammonia test.

**Figure 4:**
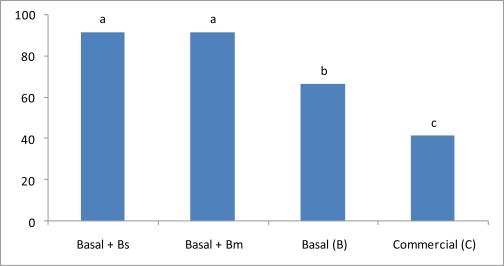
Y-axis represents percentage survival rates of *L. vannamei* during and after oxygen. Static method was tested on the different treatments. Means in the same row with different superscripts are significantly different (*P* < 0.05).

On the other hand, oxygen static test shrimps survival rate was not so drastically affected as in the ammonia test, but oxygen survival results still point out the vital importance of including probiotic feed for shrimp aquaculture (Figure 4). Treatments 1 and 2 shrimps survival rate was over 90% and did not display significant differences (*p* > 0.05). Comparing survival rates between Treatment 1 and Treatment 2, it is clear that even when *B. megaterium* did not have all *B. subtilis* probiotic capabilities, *B. megaterium* is a good candidate for improving culture oxygen concentration (Figure 4).

## 4. Discussion

Functional feed development represents one of the greatest areas of opportunity in the nutritional world. Functional feed must promote growth and health of the cultivated organisms [29], improve immune system and induce physiological benefits beyond traditional feeds [30]. In addition, functional feeds must be economically attractive to the aquaculture industry and environmentally friendly.

In this respect, animal products content in feed must be totally or partially eliminated from feed formulations and, inclusion of alternative-economical vegetable protein sources needs to be increased. Furthermore, the type and amounts of carbohydrate content is also of great concern for economical, environmental and health issues. Vegetable protein-carbohydrate well balanced functional feed could maximize benefit to animals, avoiding economical losses, environmental pollutants and diseases. However, proteases and carbohydrolases shrimp deficiency is a major impediment to digest vegetable sources limiting its high level inclusion in shrimp formulations [10]. Soybean meal antinutritional compounds and starch complex carbohydrates are good examples of undesirable-indigestible ingredients in shrimp vegetable-based feed formulations [9,15,29].

Including probiotic bacteria in feed has emerged as a new field with huge application in the aquaculture industry [17,18]. Today, *Bacillus* species are the most investigated bacteria for animal probiotic development due to: its growth nutrients versatility, high enzyme levels production and secretion of antimicrobial peptides [31–33]. In addition, *B. subtilis* is generally recognized as safe (GRAS) by the food and drug administration (FDA), meaning it is harmless to animal and humans [34,35]. In this work, we used previously characterized *Bacillus* strains as potential probiotics [10] to formulate shrimps functional feeds. Table 1 shows proximal composition of vegetable-basal feed and a fishmeal formulation. In this sense, literature indicates that optimal protein and carbohydrates concentration in shrimp feeds must be 30% and 20% respectively [25,29]. Higher protein inclusion levels are not necessary for shrimp development and could be an important aspect for water quality deterioration and cost increases. In addition, lower protein levels than recommended induce growth alteration and diseases by changing the immunological response capabilities and health of the shrimps [28,29]. Nevertheless, commercial feeds contain almost 40% FM protein increasing costs and environmental deterioration (Table 1). In this sense, 100% replacement of FM by SBM has not been successfully achieved in shrimp feeds until now; only replacement of 20% has been obtained [36]. In this work we obtained significant growth differences between shrimps fed with a commercial FM and a SBM-CHOs feed formulation (Table 1) using a specifically selected *B. subtilis* probiotic strain (Table 2). Furthermore, FCR results obtained for Treatment 1 shrimps presented significant differences compared to the other treatments (Table 2). The inclusion of a specifically selected *B. subtilis* strain in a SBM-CHOs basal-feed, transformed this formulation to an excellent alternative for shrimp nutrition (Table 2). Comparing results from Treatment 1 and Treatment 3, where *B. subtilis* was and was not included respectively (Table 2), we concluded that *B. subtilis* was the principal factor for SBM-CHOs feed digestion and assimilation. Moreover, group and specific PCR analysis demonstrated that *B. subtilis* represented almost 90% of the total bacterial diversity in shrimp intestine and feces (data not shown) [22,23]. In addition to a better growth, survival and FCR presented by Treatment 1 shrimps where *B. subtilis* was included, also; glucose, cholesterol and total hemocyte counts (THC) were higher and significantly different with respect to the other treatments (Table 3). Shrimp immune-like system and health has a strong protein-carbohydrate digestion-assimilation relationship [28]. In this sense, hemolymph metabolites like those previously mentioned could be considered direct indicators of shrimp health. These higher level hemolymph metabolites signal efficient nutrients assimilation when they concord with healthy growth, good survival and a competitive FCR (Tables 2 and 3). The THC, glucose and cholesterol levels obtained from Treatment 1 shrimps were higher and significantly different than in the other treatments. In addition, these results are strong evidence that SBM replaced FM without any negative consequence actually; betters results were obtained using SBM and *B. subtilis* (Tables 2 and 3). On the other hand, shrimp’s feed containing a high level of carbohydrates could lead to animal deficiencies and even mortality.

Pascual and coworkers demonstrated that hemolymph metabolites including lactate, glucose, proteins and immune response of the wild shrimps were better than those from cultured shrimps [15]. Essentially, they concluded that wild and farmed shrimp responded differently to diets including the same high levels of carbohydrates by cultured shrimps losing some gene expression capacity. Furthermore, Arena and coworkers in 2003 mentioned that shrimp’s limited starch digestion capacity was related to a repression of amylase alleles that, at the same time, limit dietary CHOs metabolism in general.

The concept that carbohydrates can substitute proteins as energy source in shrimp feed is already documented [37]. However, as we mentioned previously, cultured shrimps have severe restrictions for dietary CHOs utilization [15,38]. Furthermore, the inability of the shrimp’s alfa-1,4-glucosidase to cleave the alfa-1,6-bond of amylopectin resulted in low growth rates and energy waste [13,14]. In addition, melibiose, raffinose and stachyose oligosaccharides constitute 15% of SBM and are considered antinutritional compounds to shrimp development [39]. Probiotic bacteria are frequently used as feed additives to farm and pet animals. In shrimp feeds, the probiotic bacteria can facilitate digestion of protein constituents and carbohydrates, because the enzymes produced by the bacteria can complement the shrimp enzyme activity, increasing food digestibility. In addition, the probiotic enzymes have a broader pH, temperature and target range than the shrimp enzymes prolonging digestion time [10]. Table 3 indirectly shows carbohydrates degradation capacity of the *B. subtilis* probiotic enzymes. Glucose, cholesterol and THC from Treatment 1 shrimps where *B. subtilis* was included, were higher and statistically different from the other treatments (Table 3). Considering high hemolymph metabolite levels of carbohydrates with regard to probiotic digestion capacity and shrimp health, we concluded that our *B. subtilis* strain enhances CHOs digestion and assimilation and, induced a healthy condition in *L. vannamei* (Tables 2 and 3).

Lactate is the end product of glycolysis after a reduction of pyruvate by NADH [40] and, in this sense [41], showed that a high percentage of glucose metabolized during glycolysis in Crustacea (50%) is directed to form amino acids through the transamination pathway. In this sense, the almost 50% CHOs added to Treatment 1 where *B. subtilis* was included, in addition to energy production, CHOs seem to be contributing to protein generation, growth and improvement of shrimp immune system, because levels of lactate were higher and statistically different from the other treatments (Tables 2 and 3) [15,28]. Additionally, high levels of lactate start glycogen synthesis by diminishing hyperglycemic hormone (CHH) and increasing glycogen synthetase [42]. Glucose is the principal energy source in animals, and shrimps are not the exception. Energy production throughout glycolisis and Krebs cycle depends principally on CHOs type and levels in the formulation. Glycogen is an energy storage source, generated when high levels of glucose-lactate is present in shrimp hemolymph, due to adequate CHOs digestion-assimilation-metabolism processes. Glycogen-glucose reconversion throughout glycogenolisis is carried out to satisfy unexpected requirements of energy, such as during starvation, and for fast response to stress and diseases. In this sense, ammonia stress-experiments carried out in this work have shown that Treatment 1 shrimps, where *B. subtilis* was included, tolerated well high levels of ammonia throughout the 24 hrs of the experiment (Figure 3). Treatment 2, shrimps where *B. megaterium* was included, only presented a 25% survival rate (Figure 3). Different responses between Treatment 1 and 2 could be due to *B. megaterium* inducing: (a) lower levels of glucose-lactate and hence lower levels of energy source; and/or (b) by the lower capacity of *B. megaterium* to convert or transform ammonia in shrimp hemolymph and/or in water. Taking into account that Treatments 3 and 4 do not present survival data and that glucose and lactate were not statistically different between Treatments 2, 3 and 4, we can hypothesize that Treatment 2 shrimps survived by a direct ammonia conversion-transformation reaction accomplished by *B. megaterium*. It is important to remember that Treatments 3 and 4 had no *Bacillus* probiotic strains in its formulation.

In 2000 [43] reported that 100% mortality occurred at 30 mg/L of ammonia in 24 h of exposure. However, in this work, Treatment 1 shrimps, where *B. subtilis* was included to a SBM-CHOs basal feed, had an 80% survival rate on 60 mg/L of ammonia concentration; results that have not previously been reported (Figure 3). In addition, results obtained with Treatment 1 demonstrated that shrimp health condition generated by the functional feed formulated using the *B. subtilis* probiotic strain and the vegetable basal feed, was better than the other treatments (Tables 2 and 3, Figure 3). Further experiments will be conducted to specifically determine the *B. subtilis* ammonia conversion-transformation capacity. On the other hand, even when oxygen depletion stress-experiments do not present such drastic mortality results as the one presented by ammonia experiments, 35 and 60% shrimp mortality was obtained by the basal (B) and commercial feed (C), respectively (Figure 4).

In all aquatic systems, the toxicity of excreted nitrogen compounds is the most limiting parameter once adequate dissolved oxygen levels are maintained [44]. Ammonia is the main nitrogenous product excreted by crustaceans [45], and additionally is produced from the ammonification of organic matter in a culture system [46]. In this sense, basal feed formulated contains almost 25% less protein than the commercial feed (Table 1), which could be the reason why Treatment 3 (basal feed) exhibited higher shrimp survival than Treatment 4 (commercial feed)(Figure 4). On the other hand, Treatments 1 and 2 presented approx 90% shrimps survival, even when energy sources, growth and other hemolymph parameters were statistically different between both treatments. In this sense, Figure 4 results allowed hypothesizing that Treatment 2 shrimps’ positive effects was due to the accumulation of the 25% ammonia conversion-transformation capacity of *B. megaterium* (Figure 3) plus the 65% shrimp default survival presented by the basal feed (B) (Figure 4). Additionally, 90% shrimp survival in Treatment 1 could be the consequence of several factors, such as: (a) ammonia conversion-transformation by the *B. subtilis* probiotic strain; (b) less content-production of ammonia by the functional feed; (c) ammonia less oxygen consumption by the functional feed; and (d) by the extensive physiological and biochemical parameters exhibited.

## 5. Conclusions

The results obtained in this work suggest the beginning of a new era shrimp/fish functional feed formulation, where animal products inclusion in feed could be totally replaced with vegetable protein and carbohydrates sources, utilizing the appropriate probiotic bacteria. Specifically selected *B. subtilis* strains are recommended to formulate functional and economical feeds containing high levels of vegetable; protein and carbohydrates as main dietary sources in *L. vannamei* cultures.

## Figures and Tables

**Table 1:** Proximal composition of basal and commercial diets.

**Treatments (amount%)**		
**Items**	**Basal diet (B)**	**Commercial diet (C)**
**Crude Protein**	27.41	36.39
**Total Lipid**	6.46	3.98
**Carbohydrates**	49.50	38.42
**Moisture**	11.34	9.88
**Ash**	5.29	11.33

**Table 2:** Effect of treatment on survival and growth performance of shrimps.

**Items**	**Treatments**			
	**1** **Basal + Bs**	**2** **Basal + Bm**	**3** **Basal (B)**	**4** **Commercial (C)**
**Initial weight (g)**	5.96 ± 0.20 ^a^	5.81 ± 0.15 ^a^	5.98 ± 0.22 ^a^	6.06 ± 0.18 ^a^
**Final weight (g)**	10.71 ± 0.11 ^a^	9.69 ± 0.10 ^c^	9.48 ± 0.13 ^c^	10.38 ± 0.16 ^b^
**Daily Weight Gain (DWG; g/day)**	0.169 ± 0.003 ^a^	0.138 ± 0.004 ^b^	0.125 ± 0.003 ^c^	0.154 ± 0.001 ^b^
**Food conversion ratio (FCR)**	1.54 ± 0.07 ^a^	2.02 ± 0.18 ^b^	2.49 ± 0.15 ^c^	2.06 ± 0.22 ^b^
**Survival (%)**	100 ^a^	96.67 ± 3.87 ^a^	96.67 ± 3.87 ^a^	96.67 ± 3.87 ^a^

Means in the same row with different superscripts are significantly different (*P* < 0.05).

**Table 3:** Hemolymph metabolites values of shrimps fed with different diets.

	**Treatments**			
**Items**	**Basal + Bs**	**Basal + Bm**	**Basal [B]**	**Commercial [C]**
**Glucose (mmol/L)**	0.675 ± 0.02 ^a^	0.483 ± 0.03 ^b^	0.470 ± 0.02 ^b^	0.452 ± 0.03 ^b^
**Lactate (mmol/L)**	0.385 ^a^ ± 0.03 ^a^	0.273 ± 0.03 ^b^	0.261 ± 0.02 ^b^	0.249 ± 0.03 ^b^
**Total cholesterol (mmol/L)**	0.323 ± 0.08 ^a^	0.159 ± 0.01 ^b^	0.134 ± 0.07 ^b^	0.163 ± 0.03 ^b^
**Hemocytes (cell/mL)**	2.02 × 10^7^ ± 0.08 ^a^	9.83 × 10^6^ ± 0.18 ^b^	9.41 × 10^6^ ± 0.15 ^b^	9.63 × 10^6^ ± 0.12 ^b^

Means in the same row with different superscripts are significantly different (*P* < 0.05).
